# GBP2 acts as a member of the interferon signalling pathway in lupus nephritis

**DOI:** 10.1186/s12865-022-00520-5

**Published:** 2022-09-17

**Authors:** Yuan Zhang, Yinping Liao, Qing Hang, Dong Sun, Ya Liu

**Affiliations:** Department of Nephrology, The Affiliated Hospital of Xuzhou Medical University, No. 99, West Huaihai Road, Xuzhou, 221004 Jiangsu China

**Keywords:** Lupus nephritis (LN), GSEA, Interferon, Protein‐protein interaction

## Abstract

Lupus nephritis (LN) is a common and serious clinical manifestation of systemic lupus erythematosus. However, the pathogenesis of LN is not fully understood. The currently available treatments do not cure the disease and appear to have a variety of side effects in the long term. The purpose of this study was to search for key molecules involved in the LN immune response through bioinformatics techniques to provide a reference for LN-specific targeted therapy. The GSE112943 dataset was downloaded from the Gene Expression Omnibus database, and 20 of the samples were selected for analysis. In total, 2330 differentially expressed genes were screened. These genes were intersected with a list of immune genes obtained from the IMMPORT immune database to obtain 128 differentially expressed immune-related genes. Enrichment analysis showed that most of these genes were enriched in the interferon signalling pathway. Gene set enrichment analysis revealed that the sample was significantly enriched for expression of the interferon signalling pathway. Further analysis of the core gene cluster showed that nine genes, GBP2, VCAM1, ADAR, IFITM1, BST2, MX2, IRF5, OAS1 and TRIM22, were involved in the interferon signalling pathway. According to our analysis, the guanylate binding protein 2 (GBP2), interferon regulatory factor 5 and 2′-5′-oligoadenylate synthetase 1 (OAS1) genes are involved in three interferon signalling pathways. At present, we do not know whether GBP2 is associated with LN. Therefore, this study focused on the relationship between GBP2 and LN pathogenesis. We speculate that GBP2 may play a role in the pathogenesis of LN as a member of the interferon signalling pathway. Further immunohistochemical results showed that the expression of GBP2 was increased in the renal tissues of LN patients compared with the control group, confirming this conjecture. In conclusion, GBP2 is a member of the interferon signalling pathway that may have implications for the pathogenesis of LN and serves as a potential biomarker for LN.

**Supplementary Information:**

The online version contains supplementary material available at 10.1186/s12865-022-00520-5.

## Introduction

SLE is a common chronic, multisystem, autoimmune disease of unknown aetiology, the causes of which may include environmental and stochastic factors and genetic susceptibility [1, 2]. LN is a common and severe clinical manifestation of SLE [3–5] and a major risk factor for mortality [6]. Approximately 31–48% of patients with SLE develop LN. Furthermore, 7–31% of SLE patients are diagnosed with LN at the time of SLE diagnosis [7–9]; of these patients, 10% progress to end-stage renal disease (ESRD)[4, 5]. Patients with LN have a higher risk of death than the general population, and the risk of death is further increased if LN progresses to end-stage renal disease [10].

Previous studies on the pathogenesis of LN have focused on the adaptive immune system. It is usually assumed that lymphocyte abnormalities are the main cause of autoimmunity due to the recognition and processing of autoantigens by the immune system. These autoantigens activate the IFN-I signalling system and the resulting immune response in an organism by a series of antibodies that recognize autoantigens [11, 12]. There is evidence that the clinical manifestations of SLE are caused by biological responses triggered by the overproduction of IFN-I [13]. A variety of immune cells are affected by IFN-I, as IFN-I regulates intermediate signalling substances required for multiple cytokine responses [14]. IFN-stimulated genes (ISGs) are induced by IFN-I expression, and increased expression of IFN-inducible protein (IIP) in immune cells and its altered function can promote SLE disease progression [15]. Glucocorticoids were previously reported to improve patient survival, but treatment outcomes remain unsatisfactory [16, 17]. Although immunosuppressive therapy may alleviate this disease, recurrent episodes of the disease continue to damage the kidneys and eventually progress to ESRD [18]. In this context, there is an extremely urgent need to explore new therapies that are more effective, more targeted, and safer. Therefore, further exploration of the aetiology and pathogenesis of LN is necessary to find specific drugs for the effective treatment of this disease and to further improve the survival of patients.

In recent years, bioinformatics and microarray technologies have rapidly developed. Furthermore, bioinformatics has been widely used to identify disease-related genes and analyse disease pathogenesis, helping to identify important molecules associated with diseases and their mechanisms of action [19–21]. In this study, we used a bioinformatics approach to analyse genetic data from LN kidney tissues and normal control kidney tissues to screen for differentially expressed genes (DEGs) in LN. The aim of this study was to identify biomarkers associated with LN disease and to explore their pathways of action.

## Results

### Screening of 128 differentially expressed immune-related genes

**Fig. 1:**
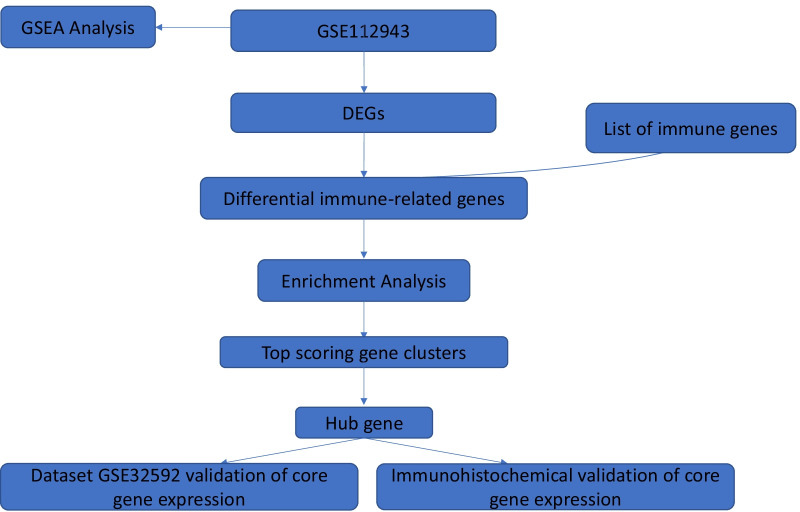
Study workflow diagram. DEGs: differentially expressed genes. GSEA: gene set enrichment analysis

**Fig. 2:**
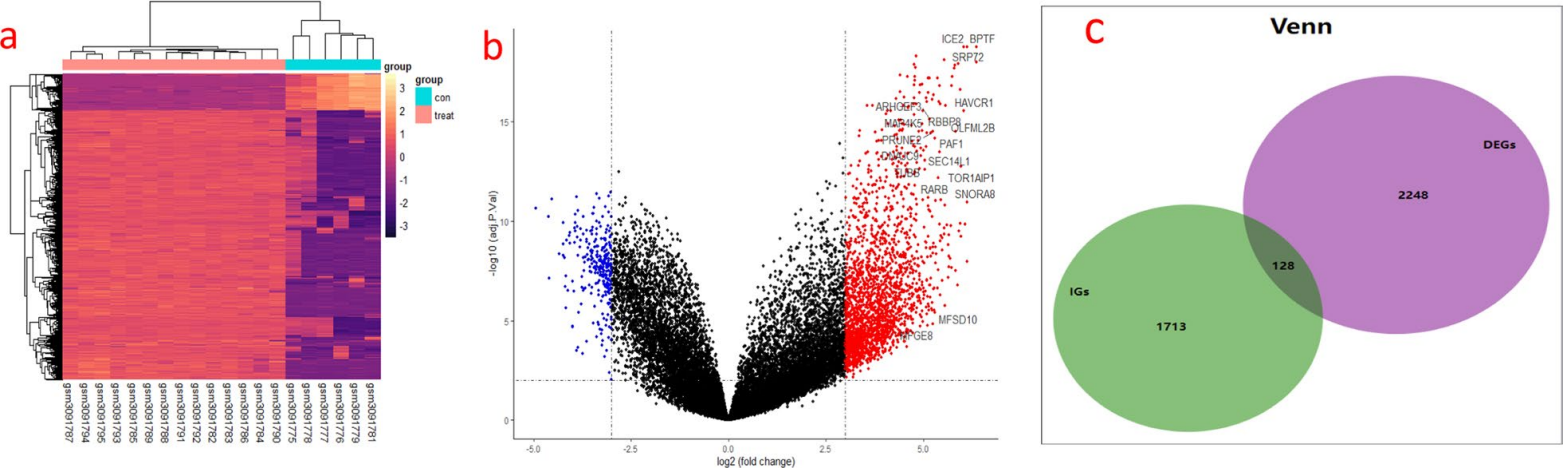
**a** Heat map of differentially expressed genes between LN and HC samples. Red rectangles indicate high expression and purple rectangles indicate low expression. hc: healthy control. **b** Volcano plot showing differentially expressed genes in LN and HC samples, blue dots represent genes significantly down-regulated in the samples and red dotes represent genes significantly up-regulated. hc: healthy control group. **c** Venn diagram showing 128 differentially immune-related genes obtained by intersecting differentially expressed genes and immune genes. DEGs: differentially expressed genes. IGs: immune genes

Figure 1 shows the specific databases used in this study and the complete workflow. Based on the sample information, 2330 differentially expressed genes were extracted from the LN samples, of which 2053 genes were upregulated, and 277 genes were downregulated. The screening criteria for differentially expressed genes were as follows: the fold change between the LN group and the control group was at least eightfold, and the corrected P value was < 0.05. To better display these differentially expressed genes, a heatmap and volcano map were drawn using R language. Heatmaps were created using the pheatmap package (Fig. 2a), and volcano plots were created using the ggplot2 package (Fig. 2b). These differentially expressed genes intersected with the list of immune genes obtained from the IMMPORT immune database to obtain 128 differentially expressed immune-related genes (Fig. 2c). These genes included 111 upregulated genes and 17 downregulated genes (Table 1).

**Table 1:** Differential immune-related genes

Symbol	Up/Down	Count
ITGAV,TLR4,ECD,RARB,HLA-DQA1,SLC40A1,MAP2K1,LYZ,IL18R1,TRIM22,PLSCR1,THRA, CBLB,GBP2,CRLF3,NFYA,C3,FGF12,PTGDR,HLA-DRA,HCK,SEMA6A,FGF11,IL7R,IFITM1, TNFRSF25,CANX,PDGFRA,DHX58,IL10RA,PIK3CG,VAV3,CCL19,TNFRSF13B,NFAT5,AGER,NRG1,SLC22A17,EIF2AK2,CTSS,RAF1,IREB2,AKT2,CXCR6,SRC,PSMC6,CSF1R,SEMA4D, LYN,GSK3B,JAG2,EGFR,NEDD4,NR2F2,CCL2,ITGAL,FCER1G,TNFRSF11B,PTPN6,IKBKG, MR1,OAS1,ADIPOR2,TAP1,TLR3,CTSG,PRKCQ,ACKR2,PSMC2,IL34,PPP3CB,KITLG, CD3D, TINAGL1,APOBEC3A,APLNR,IRF7,CMKLR1,IRF5,FIGNL2,PTPRC,S100A9,MX2,JAK1,SOS1, IL6ST,RORA,TRPC4AP,B2M,HSPA5,VCAM1,RFX5,RFXANK,MAPT,FGFR1,OSMR,IL13RA1, NR2F1,PDGFRB,HSPA8,STAT1,ENG,TYK2,ADRM1,MMP9,SEMA3B,ADAR,GCGR,SDC3, IGF2R,CXCL16	Up	111
CXCR2,SFTPD, NPR1, NMB, BMP7, BST2, MCHR2, TAFA5, IL1R2, SSTR1, SEMA5A, AVPR2, RBP2, IL7, S100A14, SEMA4C,AEN	Down	17

### Differentially expressed immune-related genes are significantly enriched in the interferon signalling pathway

**Fig. 3:**
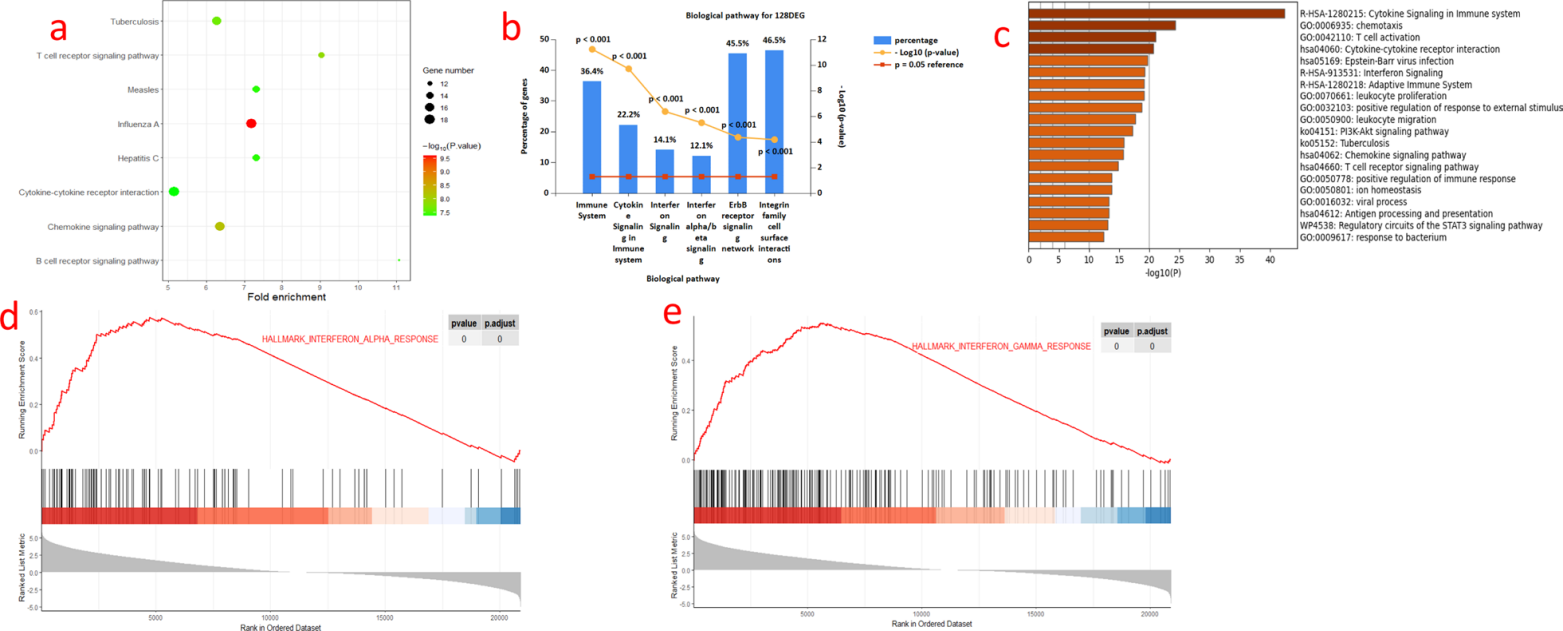
**a** Enrichment analysis of differential immune-related genes using DAVID software, with the top 8 biological pathways selected based on enrichment scores, shown using bubble plots. P < 0.05 is statistically significant. **b** Enrichment analysis of differential immune-associated genes using Funrich software, with the top 6 biological pathways selected based on P-value and gene percentage, shown using bar graphs. P < 0.05 was statistically significant. **c** Validation of the results of enrichment analysis of the differential immune-related genes using metascape software, a total of 20 pathways were enriched, shown using bar graphs. p < 0.05 was statistically significant. **d** Hallmarks gene set base used to analyse the entire gene expression values of LN and HC smaples. Significant enrichment in the interferon alpha pathway is shown, p < 0.05. **e** Hallmarks gene set database used to analyze the entire gene expression value of LN and HC samples. Shows significant enrichment in the interferon gamma pathway. p < 0.05

To identify the signalling pathways with which these 128 differentially expressed immune-related genes are involved, we further analysed the pathways enriched in these genes. The results of the DAVID software enrichment analysis showed that these genes are mainly involved in the immune response, signal transduction and microbial infection pathways (Fig. 3a). The results of the FUNRICH enrichment analysis showed that these genes were mainly enriched in the interferon signalling pathway, cytokines and the cell membrane signalling pathway (Fig. 3b).

Finally, the enrichment results were validated using METASCAPE software. The results revealed 20 pathways, including the cytokine signalling pathway, activation of immune cells and interferon signalling pathway in the immune system (Fig. 3c). The enrichment analysis results showed that these genes were significantly enriched in the interferon signalling pathway.

### The interferon signalling pathway is significantly enriched

To understand the overall gene expression, we performed GSEA for all gene expression information of the LN group and the control group using the CLUSTERPROFILER [22] software package based on the hallmark and KEGG gene set databases [23]. The default value for significantly enriched gene sets was set to a corrected P value < 0.05. GSEA revealed that the sample expression information was significantly enriched in the interferon α/γ response (Fig. 3d-e).

### PPI networks reveal four core clusters of differentially expressed immune-associated genes.

**Fig. 4:**
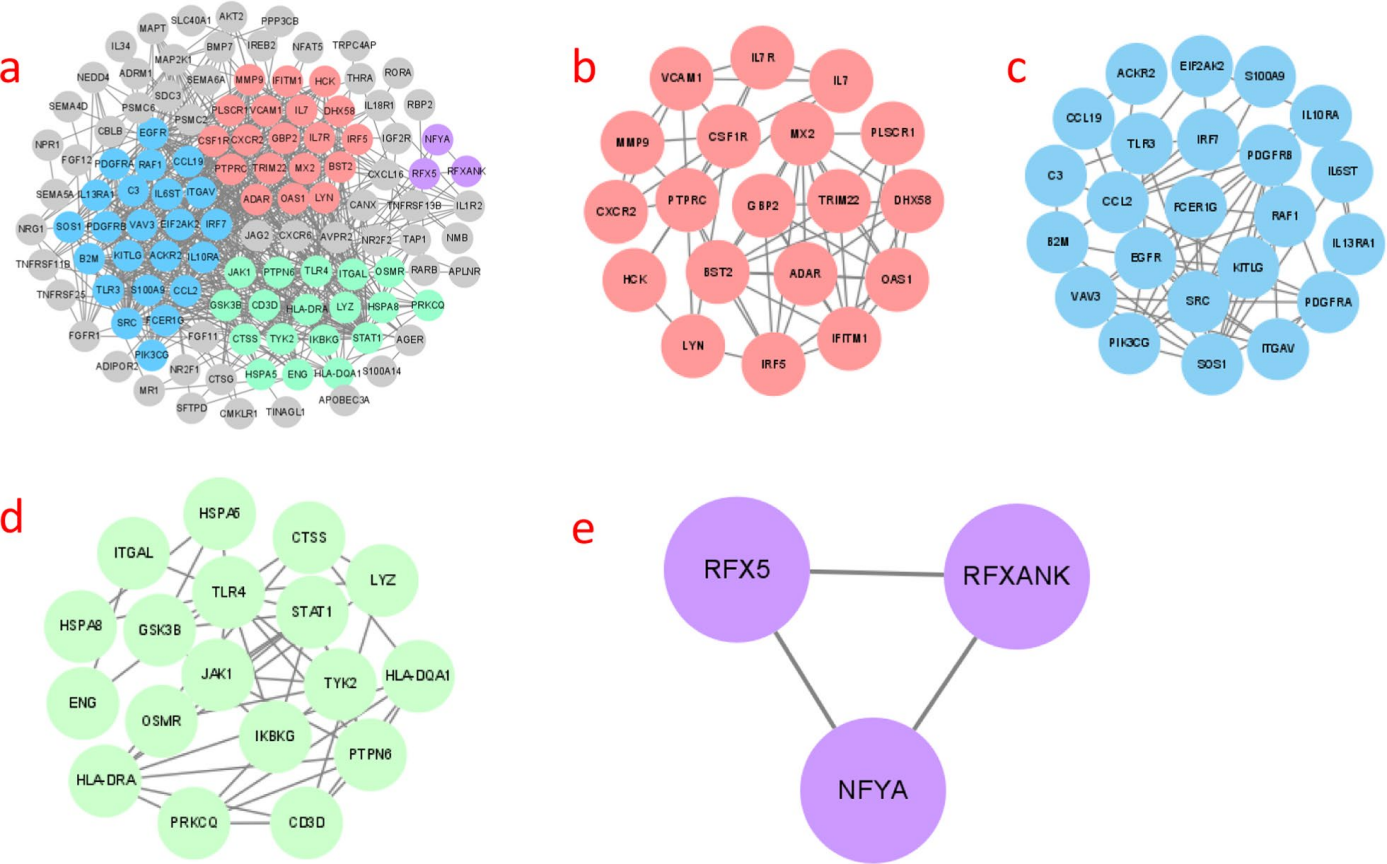
Processing the protein interaction network with Cytoscape v3.8.2. **a** The difference clusters of the MCODE analysis are indicated by different colors. **b **Gene cluster 1 (score: 6.667, 19 nodes, 120 edges). **c** Gene cluster 2 (score: 6.364, 23 nodes, 140 edges). **d** Gene cluster 3 (score: 5.294, 18 nodes, 90 edges). **e** Gene cluster 4 (score: 3, 3 nodes, 6 edges)

We further searched for core gene clusters among these 128 differentially expressed immune-related genes and analysed these genes using STRING to obtain protein interaction network maps. The network maps were visualized by CYTOSCAPE. The network map generated 116 nodes and 664 connecting lines (Fig. 4a). Data were processed with the MCODE (degree cut-off = 2, node score cut-off = 0.2, k-core = 2, maximum depth = 100) plugin to select the gene clusters (Table 2). Among these clusters, 4 gene clusters were obtained (Fig. 4b-e).

**Table 2:** The specific data of gene clusters are listed in the table below

Cluster	Score (Density*#Nodes)	Nodes	Edges	Node IDs
1	6.667	19	120	TRIM22, MX2, CXCR2, BST2, PLSCR1, DHX58, ADAR, PTPRC, LYN, IL7R, VCAM1, CSF1R, IL7, OAS1, IFITM1, HCK, GBP2, MMP9, IRF5
2	6.364	23	140	PDGFRA,VAV3,S100A9,EIF2AK2,TLR3,IL13RA1,EGFR,ITGAV,IL6ST,CCL19,KITLG,SRC, PIK3CG,IRF7,CCL2,ACKR2,C3,FCER1G, RAF1, SOS1, B2M,PDGFRB,IL10RA
3	5.294	18	90	CTSS, OSMR, JAK1, HLA-DQA1, IKBKG, HSPA8, HSPA5, TYK2, ENG, CD3D, ITGAL, PRKCQ, GSK3B, LYZ, PTPN6, HLA-DRA, TLR4, STAT1
4	3	3	6	RFXANK, RFX5, NFYA

### GBP2, IRF5 and OAS1 are involved in three interferon signalling pathways

**Fig. 5:**
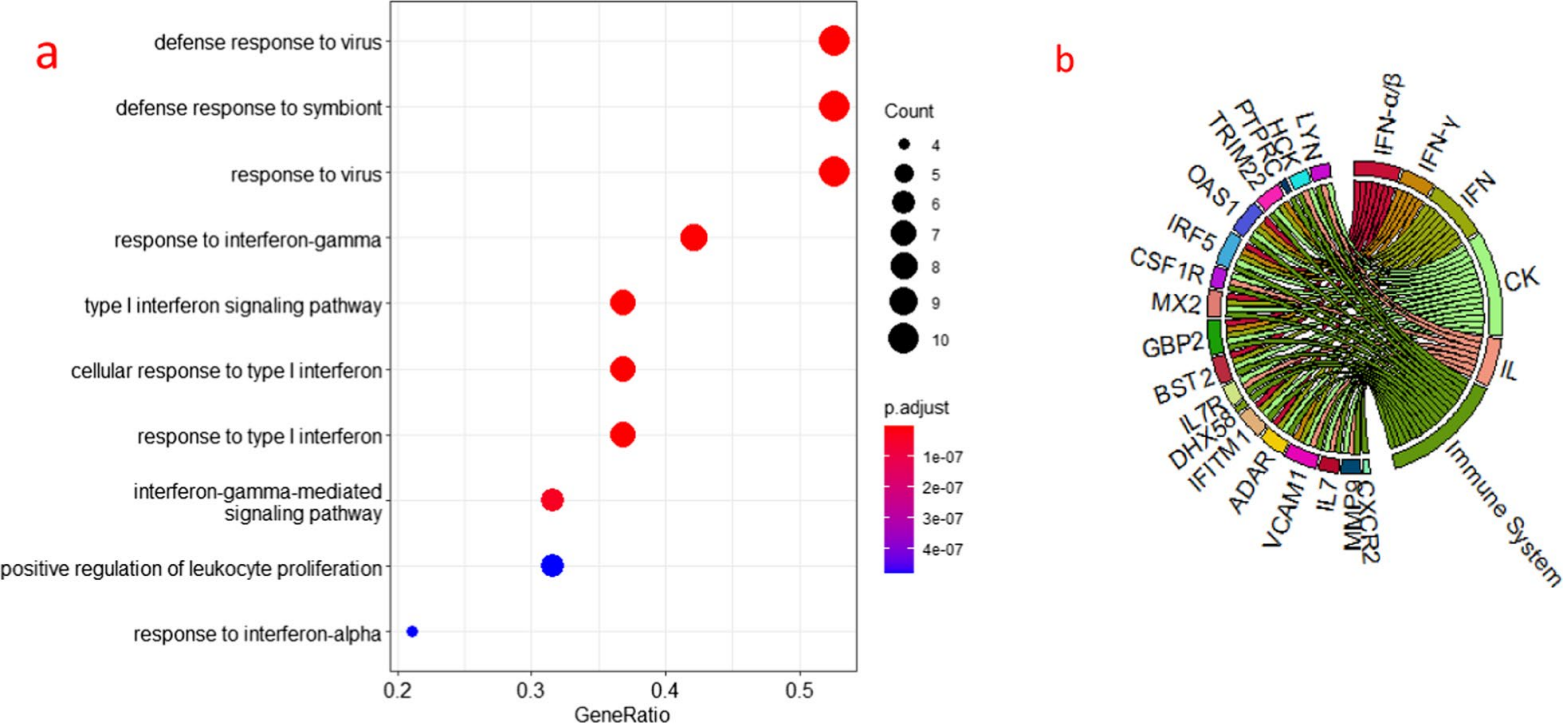
**a** GO analysis of the highest scoring gene clusters using the R language cluster Profiler package, which showed that these genes are mainly involved in biological processes such as defense responses to viruses and the interferon signalling pathway. **b** Reactome pathway results showing genes involved in the interferon signaling path way using STRING for the highest scoring gene clusters. The figures shows the genes involved in each pathway

We selected the highest scoring gene clusters for analysis to find the core genes among them. The results of GO analysis showed that these genes are mainly involved in defence responses to viruses and in the biological processes of the interferon signalling pathway (Fig. 5a). The results of STRING analysis showed that nine genes, GBP2, VCAM1, ADAR, IFITM1, BST2, MX2, IRF5, OAS1 and TRIM22, are involved in the interferon signalling pathway, three of which (GBP2, IRF5 and OAS1) are involved in three interferon signalling pathways (Fig. 5b). IRF5 is known to induce IFN expression and plays an important role in the pathogenesis of SLE [22, 23] [23, 24]. The literature also reports that OAS1 is associated with the pathogenesis of SLE [25] [26]. However, it is not clear whether GBP2 is associated with LN disease.

### GBP2 expression increases in LN in the GSE32592 dataset

**Fig. 6:**
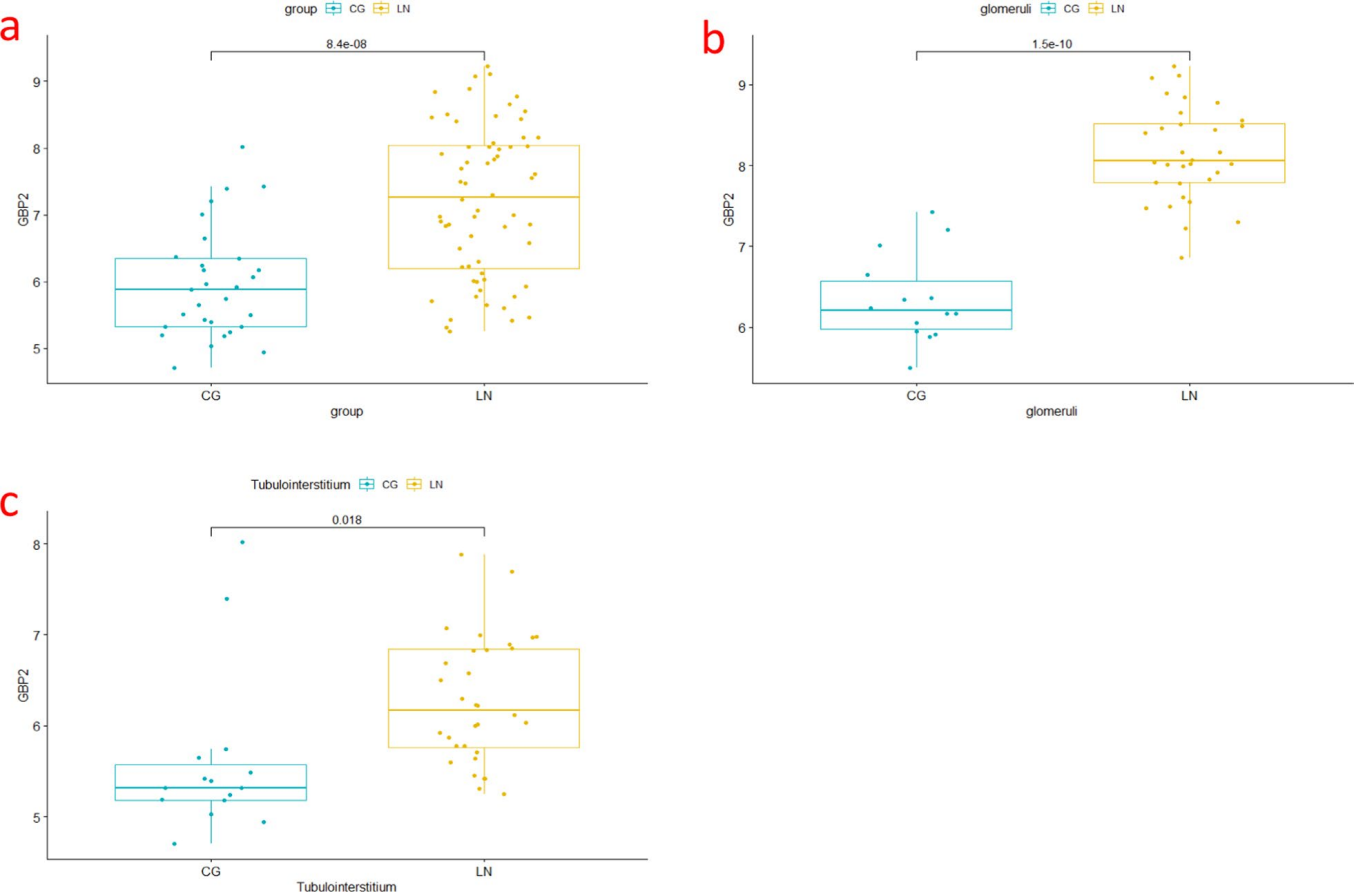
Expression of GBP2 in dataset GSE32592. **a** Expression in the kidney tissue. **b** expression in the glomeruli. **c** expression in the tubulointerstitium

To clarify the expression of GBP2 in LN, we used the GSE32592 dataset for analysis. Based on GSE32592, we first analysed the overall expression of GBP2 in kidney tissues, and the results showed that its expression was increased in LN compared with normal kidney tissues (Fig. 6a). Then, we verified the expression of GBP2 in glomeruli and tubulointerstitium, and the results also showed that the expression of GBP2 was increased in LN (Fig. 6b-c).

### GBP2 expression is significantly higher in the LN group than in the control group

**Fig. 7:**
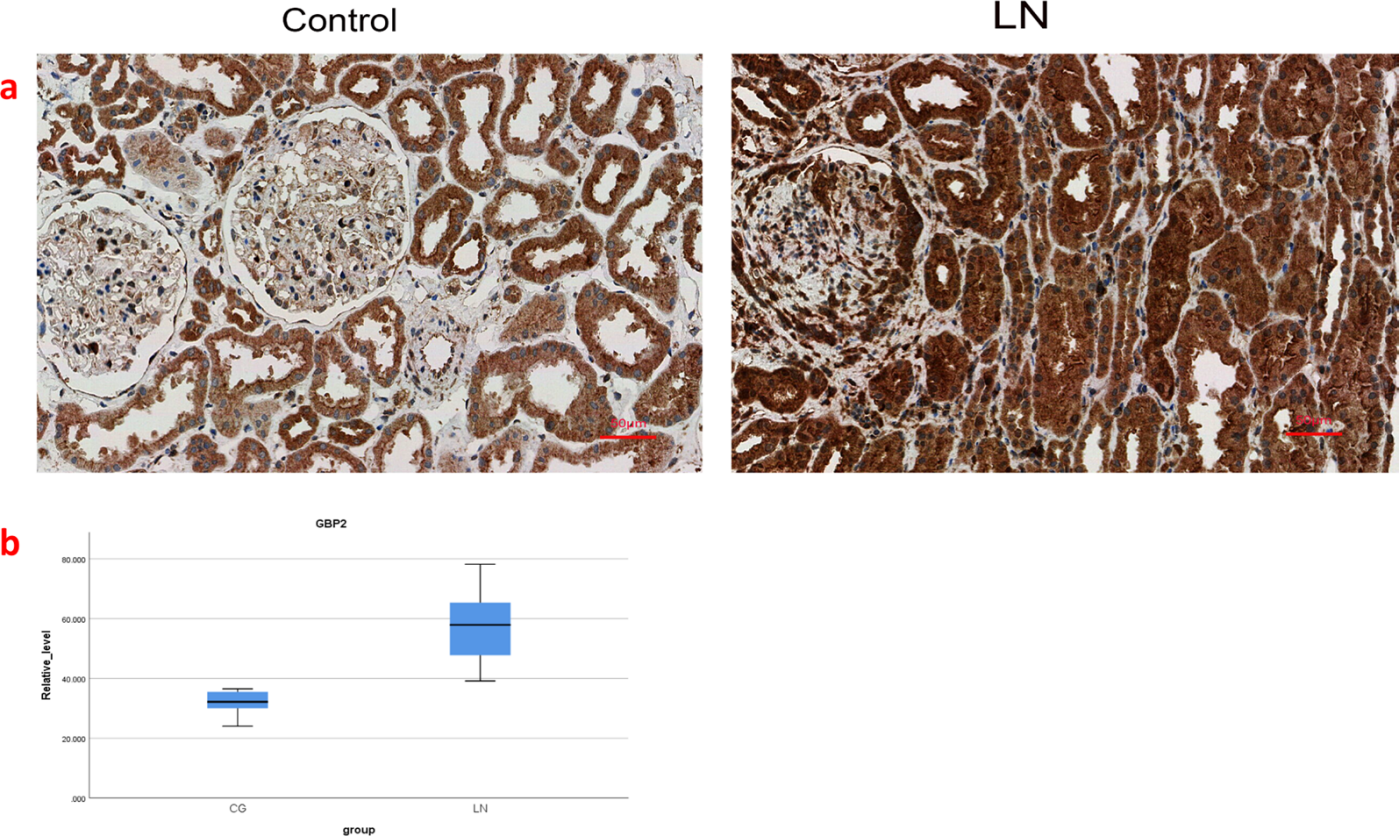
**a** Expression of GBP2 in LN and control. **b** GBP2 expression box plots for the LN and control groups, showing a statistical difference in difference in overall means (difference 25.565, CI 28.565, CI 19.773–31.358, P < 0.001)

To further validate the expression of GBP2 in LN, we analysed the expression of GBP2 in the LN group versus the control group using immunohistochemistry (Fig. 7a). Consistent with this prediction, the results showed that the expression of GBP2 in the LN group was significantly higher than that in the control group (Fig. 7b). The overall mean GBP2 expression was significantly different between the LN group and control groups (difference 25.565, CI 19.773–31.358, P < 0.001). The detailed clinical information of the patient was shown in Additional file 1: Table S1.

## Discussion

SLE is a chronic autoimmune disease that is characterized by multiple autoantibodies and involves both the innate and adaptive immune systems [27]. LN is a common clinical presentation of SLE [3–5], and its pathogenesis includes autoantibody production, abnormal activation of innate and adaptive immune responses, and immune-mediated renal injury [28]. Early studies on the pathogenesis of LN focused on the adaptive immune system. However, the molecular mechanisms underlying the pathogenesis of LN are still not fully understood, and no specific drugs have been identified to effectively treat this disease.

In recent years, the discovery of several molecules closely related to the pathogenesis of LN has greatly contributed to a new understanding of the disease and new therapeutic directions. However, the pathogenesis is still not fully understood at the molecular level, which has greatly hindered new advances in therapeutic approaches to the disease. With the development of single-cell sequencing technology and bioinformatics technology, an increasing number of genes associated with the pathogenesis of LN have been discovered [29, 30]. These genes offer the possibility to explore new targets for LN therapy.

In this study, we screened the differentially expressed genes from a dataset downloaded from the GEO database and analysed their intersection with the immune genes downloaded from the IMMPORT database. Overall, 128 differentially expressed immune-related genes were screened, including 111 upregulated genes and 17 downregulated genes. We used the DAVID, FUNRICH, and METASCAPE databases for enrichment analysis of the 128 differentially expressed immune-related genes, and we found that the interferon signalling pathway was significantly enriched. To validate the results, we analysed the overall expression information of the samples by the CLUSTERPROFILER package in R language, and the results showed that the overall expression was also significantly enriched in the interferon signalling pathway.

Persistent overexpression of interferon and its continuous stimulation of the immune system are responsible for various clinical manifestations of SLE [31]. Previous studies have shown that activation of the IFN signalling pathway is associated with LN [32] and active LN [33]. Studies have also shown that a large number of genes are regulated by interferon [31] and that LN kidney biopsies show increased expression of IFN-induced genes [34, 35].

To determine which of these 128 genes are involved in the interferon pathway, we analysed these 128 genes with MCODE and obtained the highest scoring gene cluster. We analysed this gene cluster with R software to determine the biological processes with which these genes are involved. The results showed that these genes were significantly enriched in the interferon signalling pathway. REACTOME pathway analysis showed that the genes involved in the interferon signalling pathway included nine genes: GBP2, VCAM1, ADAR, IFITM1, BST2, MX2, IRF5, OAS1 and TRIM22. Among these genes, GBP2, IRF5 and OAS1 are involved in three interferon signalling pathways. IRF5 plays a role in the pathogenesis of SLE in a variety of cells [36, 37], and OAS1 is associated with SLE pathogenesis [25]. Here, we explored the relationship between GBP2 and the pathogenesis of LN.

Guanylate binding proteins (GBPs) are IFN-inducible proteins [38]. Previous studies have shown that GBPs are mainly involved in the innate immune response to bacterial infections [39] and have an important role in protective immunity against bacterial infections [40–44]. Additionally, GBPs have a wide range of antiviral properties and play an important role in host resistance to viral infections [45]. GBP2 is a member of the GBP family. IFN-α/β and IFN-γ induce the production of GBP2 [46], which also plays an important role in resistance to infection by intracellular pathogens [47]. GBP2 inhibits a variety of viruses, including human immunodeficiency virus, hepatitis C, swine fever, Zika virus, measles, and influenza A [48–51]. GBP2 has been reported to induce cytoplasmic lysis and DNA release during bacterial infection and to promote the activation of melanoma infection factor 2 (AIM2) by the inflammasome [43].

AIM2 was first identified in melanoma [52] and is an interferon-inducible protein [53]. AIM2 has been shown to act as a cytoplasmic double-stranded DNA sensor. It is a component of the inflammasome that recognizes pathogen-associated or host-derived cytoplasmic double-stranded DNA. This triggers the production of interleukin 18 (IL-18) and interleukin 1-beta (IL-1β) and initiates the innate immune system [54, 55]. It has been reported in the literature that AIM2 may act as an important cytoplasmic double-stranded DNA sensor that induces the functional maturation of macrophages and serves as a potential biomarker for SLE disease [56].

Macrophages secrete a variety of cytokines; through these cytokines, they participate in the inflammatory response and regulate adaptive immunity [57]. Studies have shown that SLE patients present with abnormal cell death, including apoptosis, cell necrosis and enhanced autophagy, along with reduced clearance of dead cells [58]. It has been reported that macrophages are closely associated with poor prognosis by mediating inflammation and tissue remodelling, leading to LN tissue damage and renal macrophage infiltration [56]. These studies have demonstrated the role of macrophages in the pathogenesis of SLE. Additionally, experiments have increasingly reported a close relationship between SLE and macrophages [59, 60]. Conversely, blocking macrophage activation alleviates the progression of SLE, suggesting that apoptotic double-stranded DNA-induced macrophage activation may play an important pathogenic role in the development of SLE [56].

## Conclusion

The current study suggests that GBP2 is a member of the interferon signalling pathway. We found that it may play a role in the pathogenesis of LN. Immunohistochemical results showed that the expression of GBP2 was significantly increased in LN patients compared with controls. Therefore, we suggest that GBP2, as an interferon-inducible gene, plays a role in LN disease progression, providing a new perspective on the understanding of this disease. This novel finding lays the foundation for the study of the underlying mechanisms of LN and indicates potentially promising findings for clinical application.

## Materials and methods

### Acquisition of sample information

We obtained human LN expression profiles from the Gene Expression Omnibus database [61], from which we selected the GSE112943 dataset based on the GPL10558 platform [62]. From this dataset, 20 kidney samples were selected, including 14 LN kidney samples and 6 control kidney samples. All biological information for the selected samples was downloaded for the next step of analysis. The sample information and data used in this paper were downloaded from public databases.

### Data processing

The downloaded raw expression matrix was processed using R language to convert probe IDs into gene symbols and delete probes that could not be converted into gene symbols based on the annotation information in the platform file. When multiple probes all represented a gene symbol, the one with the highest expression was selected to represent the expression level of that gene. Differentially expressed genes were screened using the R language LIMMA package [63]. The criteria for selecting differentially expressed genes were as follows: at least eightfold change between the LN group and the control group and a corrected P value < 0.05. The obtained differential genes were intersected with the list of immune genes downloaded from the IMMPORT immune database to obtain the differentially expressed immune-related genes.

### Enrichment analysis of samples

The annotation, visualization and integrated discovery database (DAVID v6.8) [64] and the functional enrichment analysis tool (FUNRICH) v3.1.3 [65] were used for pathway analysis of the differentially expressed immune-related genes. The enrichment results were validated using METASCAPE [66] software, and the differentially expressed immune-related genes were uploaded to METASCAPE for pathway analysis. The pathway analysis aimed to identify the key pathways involved in the differentially expressed immune-related genes.

Gene set enrichment analysis (GSEA) was performed using the R language CLUSTERPROFILER package [67] for all genetic information in the LN group and the control group. This analysis is based on the expression of the overall genome of the sample rather than individual genes and therefore allows for the observation of more subtle changes in expression.

### Protein‒protein interaction (PPI) network analysis and gene cluster analysis

The screened differentially expressed immune-related genes were uploaded to STRING v11.5 [68] to obtain protein‒protein interaction network maps. The results of STRING analysis were imported into CYTOSCAPE v3.8.2 [69], and clustering analysis was performed using the Molecular Complex Detection (MCODE) plugin. The gene clusters with high scores were selected. We further analysed the biological processes with which the genes in that gene cluster were involved.

### Enrichment analysis of the highest scoring gene clusters

The genes in the highest scoring gene clusters were subjected to gene ontology (GO) analysis using the R language CLUSTERPROFILER package to analyse the biological processes with which these genes are mainly involved. Then, these genes were further analysed with STRING to identify the genes involved in the main biological processes.

### Dataset GSE32592 validates the expression of core genes in LN

We screened a potential core gene from the highest scoring gene cluster and analysed it using the GSE32592 dataset. Based on the information from GSE32592, we analysed the core genes at the overall kidney, glomerular and tubulointerstitial levels to clarify the expression of core genes in the LN.

### Immunohistochemistry

To further clarify the expression of core genes in LN, they were validated by immunohistochemistry. We selected renal pathological sections from 12 patients who attended the Affiliated Hospital of Xuzhou Medical University between January 2020 and December 2021. These patients included 6 patients with active LN (5 females and 1 male, age range 21–41 years) and 6 patients with hydremic nephritis, including 5 patients with membranous nephropathy and 1 patient with minimal change disease (5 females and 1 male, age range 25–56 years). The study was approved by the Ethics Committee of the Affiliated Hospital of Xuzhou Medical University, and all patients provided written informed consent. The selected LN patients met the American College of Rheumatology (ACR) classification criteria for SLE and had biopsy-confirmed lupus nephritis. Patients also had a mean activity index (AI) > 12 based on a modified National Institutes of Health (NIH) semiquantitative score. Six patients with LN with renal lesions were used as the experimental group, and 5 patients with membranous nephropathy and 1 patient with minimal change disease were used as the control group. Immunohistochemistry was performed by KingMed Diagnostics (Nanjing Jinyu Medical Testing Center Co., Ltd, China) to analyse the expression of GBP2 (PROTEINTECH Group, Inc.) in the experimental and control groups.

## Statistical analysis

The area ratios of the experimental and control groups were calculated using ImageJ software. SPSS Version 25.0 (SPSS, Armonk, NY, USA) was applied for statistical analysis of the data. Values are expressed as the means ± standard deviation, and normality was tested for the LN group and the control group using the Shapiro–Wilk test. Student’s t test was used when the variables were normally distributed in both groups, and the t’ test was used if the variance was not equal. Differences were considered statistically significant at P < 0.05.

## Supplementary Information

**Additional file 1:** The detailed clinical information of the patient.

## Data Availability

The data used in this study were obtained from the GEO database (GEO; http://www.ncbi.nlm.nih.gov/geo/): GSE112943.

## Abbreviations

LN: Lupus nephritis
SLE: Systemic lupus erythematosus
GEO: Gene expression omnibus
GSEA: Gene set enrichment analysis
GBP2: Guanylate binding protein 2
IRF5: Interferon regulatory factor 5
OAS1: 2′-5′-Oligoadenylate synthetase 1
ESRD: End-stage renal disease
ISG: IFN-inducible gene
IIP: IFN-inducible protein
DEGs: Differentially expressed genes
ACR: American College of Rheumatology
AI: Activity index
NIH: National Institutes of Health
AIM2: Activation of melanoma infection factor 2
IL-18: Interleukin 18
IL-1β: Interleukin 1-beta
