# Supplementary material for: GBP2 acts as a member of the interferon signalling pathway in lupus nephritis
Source: BMC Immunol. 2022 Sep 17;23:44. doi: 10.1186/s12865-022-00520-5 (PMC9482746; doi:10.1186/s12865-022-00520-5)
Supplement: Supplementary file 1 — Additional file 1: The detailed clinical information of the patient. [file 12865_2022_520_MOESM1_ESM.docx]

**Supplementary 1**: The detailed clinical information of the patient.

| **No.** | **Sex** | **Age(y)** | **Clinical Diagnosis** | **Pathological Diagnosis** |
| --- | --- | --- | --- | --- |
| 1 | Female | 39 | Lupus Nephritis | Lupus Nephritis |
| 2 | Male | 37 | Lupus Nephritis | Lupus Nephritis |
| 3 | Female | 28 | Lupus Nephritis | Lupus Nephritis |
| 4 | Female | 25 | Lupus Nephritis | Lupus Nephritis |
| 5 | Female | 21 | Lupus Nephritis | Lupus Nephritis |
| 6 | Female | 41 | Lupus Nephritis | Lupus Nephritis |
| 7 | Male | 39 | Membranous Nephropathy | Membranous Nephropathy |
| 8 | Female | 51 | Membranous Nephropathy | Membranous Nephropathy |
| 9 | Female | 46 | Membranous Nephropathy | Membranous Nephropathy |
| 10 | Female | 49 | Membranous Nephropathy | Membranous Nephropathy |
| 11 | Female | 56 | Membranous Nephropathy | Membranous Nephropathy |
| 12 | Female | 25 | Minimal Change Disease | Minimal Change Disease |
